# Case report: Findings in ovaries development from an aborted equine fetus

**DOI:** 10.3389/fvets.2024.1275220

**Published:** 2024-05-16

**Authors:** Matteo Cuccato, Andrea Bertuglia, Sara Divari, Eleonora Brambilla, Valeria Grieco, Enrico Bollo, Frine Eleonora Scaglione

**Affiliations:** ^1^Department of Veterinary Sciences, University of Turin, Turin, Italy; ^2^Department of Veterinary Medicine and Animal Science, University of Milan, Lodi, Italy

**Keywords:** foal, hamartoma, ovary, neoplasia, abortion, fetus

## Abstract

An aborted female foal was submitted for necropsy. During the gross examination, the ovaries were pale, grayish, and enlarged (6 × 5 cm), with a well-developed vascular structure surrounding the external surface; the cut surface of the ovaries showed a brownish parenchyma with white follicular areas mainly localized in the peripheral region. The ovaries were fixed for histological investigations. The histological evaluation of the ovaries showed polygonal-shaped cells with abundant cytoplasm and round or oval nuclei, arranged in cords of single cells. The tissue architecture was characterized by the presence of lobular-like tissues with a central vein. The tissue mimicking hepatocytes was delimited by a mature fibrous tissue and was surrounded by the normal ovarian tissue characterized by germinal epithelium and primordial follicular structures. Based on the histological findings, a diagnosis of bilateral ovarian hamartoma was carried out initially. For a better characterization of the ovarian tissue, the expression of tissue-specific (liver and ovary) markers was investigated using immunohistochemistry. Following the immunohistochemical analysis, the hamartoma diagnosis was excluded. The ovaries exhibited unique characteristics different from those of adult horse ovaries as well as unique morphological features different from other mammalian species. This case report enhances our understanding of ovaries at a later stage of pregnancy and unveils unique characteristics of horse ovaries development, avoiding misdiagnosis with pathological findings, hamartomas, or neoplasia.

## 1 Introduction

Understanding the normal organ development during embryonic and fetal stages is crucial for identifying physiological or abnormal findings in both pregnancy and adulthood (1). While there have been significant contributions to the general development of genital organs linked to the urinary system in various mammals, detailed chronological information on gonadal organogenesis in male and female embryos and fetuses is lacking for many species, including horses (2). In horses, a distinctive feature in fetal genital tract development is gonadal hyperplasia, commonly observed from mid-pregnancy onward, but possibly starting earlier (2, 3). Fetal gonadal hyperplasia (testes or ovaries) during the intrauterine phase leads to elevated estrogen levels in the mother (4). Between 240 and 270 days of gestation, the fetal ovary can be twice the size of the adult mare's ovary, occupying approximately one-third of the abdominal cavity (4). Toward the end of gestation, the gonads' size decreases, resulting in a reduction in maternal estrogen levels (4, 5). On the other hand, an increase in the size of fetal ovaries during pregnancy or in newborn foals may be a finding suggesting the presence of a congenital neoplasia. In fact, congenital tumors affecting the female reproductive system, though rarely observed, are identified in newborn or young foals (6, 7). A literature review reveals that both malignant and tumor-like lesions occur in female foals. Malignant tumors are more commonly observed in adult mares than in young foals, with granulosa cell tumors being the predominant diagnosed neoplasia (8). Nonetheless, rare neoplasms are documented, including a case of metastatic ovarian teratocarcinoma in a 3-years-old Quarter Horse mare (9). Conversely, ovarian teratomas are the most prevalent non-malignant congenital lesions reported in the reproductive system of young female foals and occasionally manifest as clinical findings at a later age (6–8, 10, 11). Teratomas are non-malignant neoplasms composed of the derivates of at least two of the embryonic layers: endoderm, mesoderm, and ectoderm (including neuroectoderm), exhibiting varying levels of maturation. In contrast, hamartomas are non-neoplastic proliferations of mature and well-differentiated tissue in an atypical anatomical location (12). These congenital lesions, less extensively documented than teratomas, include a limited number of cases describing ovarian hamartomas with an interstitial cell population in young foals or mares (13, 14). The main aim of this short communication is to describe an unusual bilateral ovarian presentation in an aborted foal and to characterize this ovarian finding.

## 2 Materials and methods

### 2.1 Case presentation

In February 2023, a female fetus was aborted at 10 months of gestation by a 12-year-old healthy Italian trotter mare. During her life, the mare had only one pregnancy that she carried to term, resulting in the delivery of a healthy foal. In her second pregnancy, a regular prophylaxis for EHV1, EHV4, EI, and WND was applied, and an American trotter stallion's semen was selected for insemination. During previous examinations, placentitis signs were never observed and the abortion was unexpected. The fetus appeared of normal size and the placenta was regularly expelled; however, umbilical cord torsion was observed at the time of abortion.

### 2.2 Necropsy and tissue sampling

The aborted female fetus was submitted for necropsy at the Department of Veterinary Sciences in Turin. The ovary and uterus were collected and fixed in 4% buffered formalin. In addition, the placenta, the liver, kidneys, spleen, lungs, and a long bone were collected for microbiological investigations. Formalin-fixed organs were processed for histopathology with H&E staining.

### 2.3 Immunohistochemistry

In addition to the H&E morphological evaluation, tissue-specific immunohistochemistry (IHC) was conducted. In particular, ovary and liver-specific antibodies were tested using IHC on the collected horse tissues. For the liver, anti-hepatocyte antibody (1:50 dilution in PBS, mouse monoclonal antibody specific to human hepatocyte, clone OCH1E5, M7158, DAKO) was tested, following already described methods (15). The anti-Müllerian hormone antibody (1:500 in PBS, mouse monoclonal antibody specific, SantaCruz) was used as an ovarian specific marker already tested in the mare (16). Equine liver and ovary tissues from archival FFPE samples of the Department of Veterinary Sciences in Turin were used as positive and negative controls.

## 3 Results

### 3.1 Gross evaluation

**Figure 1:**
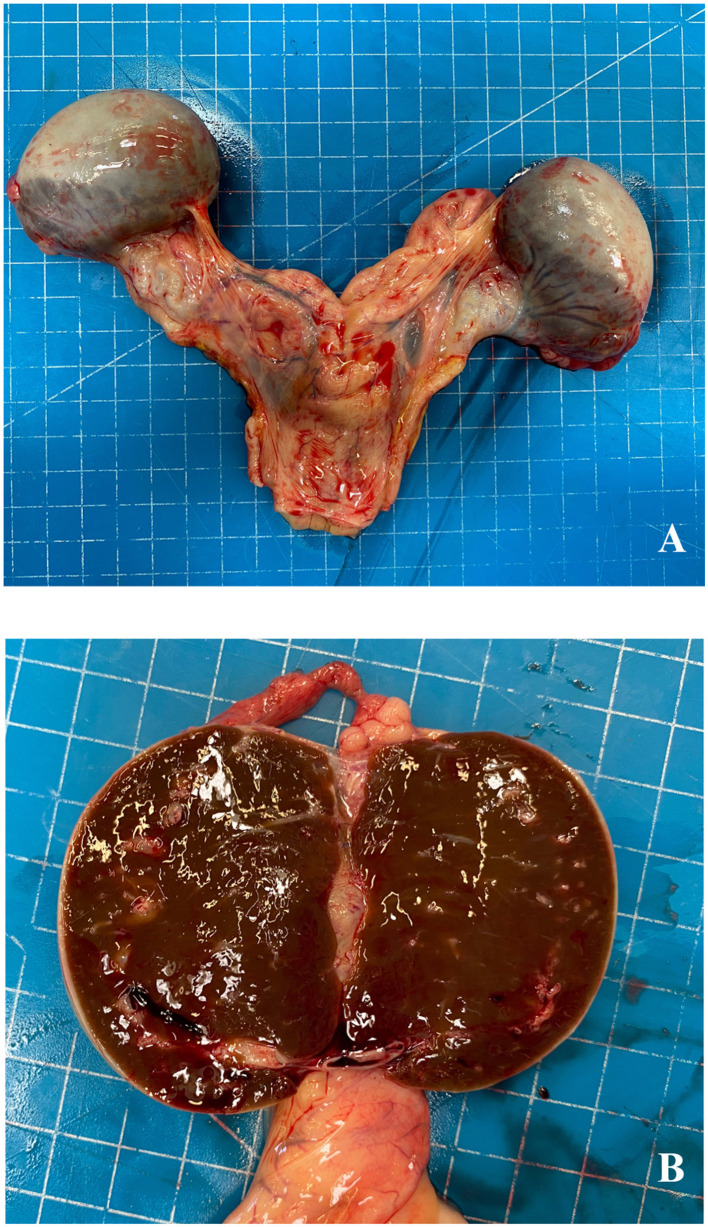
Aborted fetus, ovaries. External macroscopic aspect of ovaries **(A)** and at the cut surface **(B)**.

At necropsy, the lungs and the heart were characterized by diffuse petechiae and acute renal strokes, and thickening of the pyloric region was also detected. The ovaries were pale, grayish, and enlarged (6 × 5 cm), with a well-developed vascular structure surrounding the external surface (Figure 1A); the cut surface of the ovaries showed a brownish parenchyma with white follicular areas mainly localized in the dorsal region (Figure 1B).

### 3.2 Histopathological evaluation

**Figure 2:**
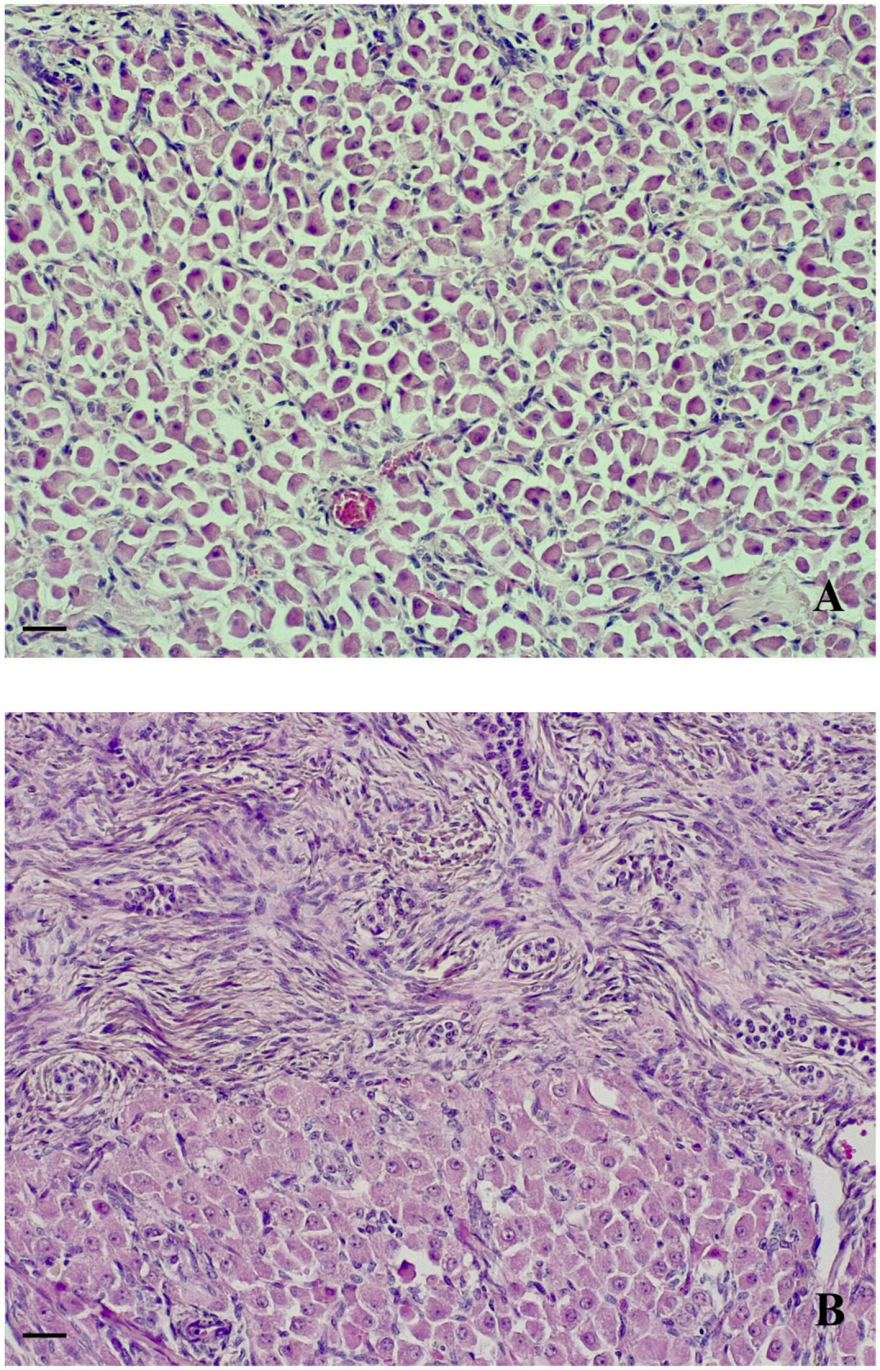
Aborted fetus, ovaries. Polygonally shaped cells arranged in cords **(A)**. The tissue is delimited by mature fibrous tissue and surrounded by germinal epithelium and follicular structures **(B)** (HE, 200 × , scale bar 500 μm).

Histological evaluation of ovaries showed polygonal-shaped cells with abundant cytoplasm and round or oval nuclei, arranged in cords of single cells (Figure 2A). The tissue architecture was characterized by the presence of lobular-like structures with a central vein. The sampled tissue was delimited by the mature fibrous tissue and surrounded by the normal ovarian tissue characterized by germinal epithelium and primordial follicular structures (Figure 2B). Finally, the uterus presented a normally developed histologic structure.

### 3.3 Immunohistochemistry

The collected sample tested negative for the expression of hepatocyte and ovarian markers. No specific signal was detected for the expression of the investigated markers.

## 4 Discussion

According to extensive literature research on horse hamartomas, only three cases with a diagnosis of hamartomas in the liver (17) and the ovary (13, 14) have been reported in aborted equine fetuses. In particular, the case presentation and diagnosis were similar between the above-mentioned published case reports and the present one. In all cases, the fetuses were aborted during a pre-term stage of pregnancy, or the newborn foal just died a few hours after delivery, as in the case reported in this study.

For this reason, further diagnostic investigations have been conducted, and in addition to the H&E morphological evaluation, IHC tests for hepatocytes and anti-Müllerian hormone were performed. The collected tissue, assumed as an ovarian hamartoma, was negative for both markers, excluding the hepatic differentiation and the presence of granulosa cells. Therefore, the first diagnosis of ovarian hamartoma was excluded, and the collected tissue was most likely a normal ovary of a horse fetus. In addition, the presence of ectopic hepatic tissue in ovaries was also discarded considering the negative immunohistochemistry results. However, the morphological appearance of the collected tissue was not in line with a normal adult horse ovary. The scientific literature on ovarian developmental steps of the equine fetus is limited, and the macroscopic and microscopic related images can be difficult to evaluate due to their low quality and old publication date (2). The only recent investigation on the development of horse gonads during pregnancy was conducted and published by Barreto et al. (18). According to this research, equine ovaries during pregnancy are characterized by substantial interstitial tissue hyperplasia producing high levels of estrogens. For this reason, fetal ovaries can significantly increase in size, and during their development, they can even become bigger than kidneys. During fetal development, the size of the interstitial tissue gradually decreases, and after birth, the inversion of cortical and medullary layers occurs. Nevertheless, this investigation considered a horse fetus aged between 40 and 90 days of gestation. The morphology of ovaries from older fetuses has never been reported. Considering our case report, the aborted fetus of this case report was aged approximately 300 days of gestation, and to our best knowledge, no information is available describing the morphological development of horse ovaries at later pregnancy stages.

In conclusion, in this case report, the macroscopic and microscopic evaluations were conducted on ovaries collected from an aborted foal aged ~300 days of gestation. The ovaries exhibited unique characteristics different from adult horse ovaries as well as unique morphological features different from other mammalian species. This case report enhances our understanding of ovaries at a later stage of pregnancy and unveils unique characteristics of horse ovaries development, avoiding misdiagnosis with pathological findings, hamartoma, or neoplasia.

## Data availability statement

The original contributions presented in the study are included in the article/supplementary material, further inquiries can be directed to the corresponding author.

## Ethics statement

Ethical approval was not required for the studies involving animals in accordance with the local legislation and institutional requirements because described procedures were conducted as part of the routine diagnostic service of the department. Written informed consent was obtained from the owners for the participation of their animals in this study.

## Author contributions

MC: Data curation, Formal analysis, Investigation, Methodology, Visualization, Writing original—draft. AB: Investigation, Methodology, Resources, Writing—review & editing. SD: Investigation, Methodology, Writing—review & editing. EBr: Investigation, Methodology, Writing—review & editing. VG: Investigation, Methodology, Supervision, Writing—review & editing. EBo: Writing—review & editing. FS: Conceptualization, Formal analysis, Investigation, Methodology, Project administration, Resources, Supervision, Writing—review & editing.
